# Case of Rupture of the Uterus

**Published:** 1859-07

**Authors:** 


### *Case of Rupture of the Uterus.*

To the Editor of the Buffalo Medical Journal.

*Sir:*—Should you think the following case worthy of publication, I should feel obliged by its insertion in your Journal.

At 4 o'clock A.M. of March 11th, I was summoned to meet Dr. Brenen in consultation. The subject was a Mrs. M——, in labor with the ninth child. The Doctor had been with her two hours previous to my arrival. He remarked that her pains had not been severe. I found her extremities cold; her pulse scarcely perceptible; much anxiety of countenance; oppression of breathing; pain on pressure over the abdomen. She felt acute pain in the epigastrium when it occurred. To use her own language, "Something snapped." Vomiting ensued immediately. I immediately delivered her with the forceps of a large still-born fœtus. I removed the placenta, and applied a bandage with a compress over the uterus. Stimulants were given, with opiates; but all in vain. Death closed the scene in one hour from the time I arrived.

*Postmortem.*—Present Drs. Brenen and Freeland. My first incision revealed a large quantity of blood in the peritoneal cavity. The uterus was found in a position more toward the left iliac fossa than the mesial line. The entire organ was above the pelvic brim. The uterine structure was firm and thick, but towards the cervix and os it appeared flattened and thinned. The Fallopian tubes and ovaries on each side were healthy. A laceration was found in the posterior wall of the uterus, at the junction of the cervix with body, about five inches in length.

Yours, &c.,

N. C. Husted, M. D.,

217 W. 42d St., N. Y. City.

*Changes in Medical Schools.*—We gather from our exchanges quite a budget of information in regard to Medical Colleges, in some of them the changes being quite important. We have also the news of the establishment of several new institutions: one at Mobile, in which Dr. J. C. Nott is to take Chair of Surgery, the rest of the faculty not having been announced; the University of the Pacific; and the Lind University.

The Lind University is established upon a basis so different from the other institutions for medical instruction as to demand some comment and explanation. Prof. N. S. Davis, formerly the editor of the Chicago Medical Journal, who was connected also with the Rush Medical College, withdrew, with several of the faculty, and established the Lind University upon the principle which Prof. Davis has so long advocated. Whether it will be a success remains to be seen; we regard as extremely doubtful both its success and its benefit to the cause of medical education. We have not the circular at hand, but copy from the Chicago Journal the plan of instruction.

Plan of Instruction.—Each college term will consist of two departments, essentially distinct from each other, but carried on simultaneously. The first, called the *junior department*, will embrace full courses of lectures and demonstrations on the following branches, viz.: Descriptive Anatomy, Physiology and Histology, Materia Medica and General Therapeutics, General Pathology and Public Hygiene, Inorganic Chemistry, and Practical Anatomy under the direction of the demonstrator, and is designed for all students attending the first course of lectures.

All medical students in this department will be examined at the end of the term, on the branches taught in the course; and if such examination be satisfactory, it will be final for those branches.

The second, called the *senior department*, will embrace full courses of lectures on the principles and practice of Surgery, Surgical Anatomy, Obstetrics and Diseases of Women and Children, Practice of Medicine, Organic Chemistry and Toxicology, Medical Jurisprudence, Clinical Medicine and Surgery in the Hospital, and Dissections under the demonstrator, and is designed for the students attending their second course.

The regular annual course of lectures in the medical department of the Lind University, will commence on the second Monday in October next, and end on the first Monday in March following.

Prof. George B. Wood, the eminent teacher of Practical Medicine in the University of Pennsylvania, has signified his intention of resigning, after the next session. Prof. Wood is perhaps our most distinguished man in the department of practical medicine, and known to the profession of this country and abroad chiefly by his work on Practice, and as one of the authors of the United States Dispensatory. He has also published a highly successful work on Materia Medica and Therapeutics. Prof. Wood has retired from the Pennsylvania Hospital, where he is succeeded by Dr. F. G. Smith.

We learn that the entire faculty of the Pennsylvania Medical College has retired, and that the faculty of the Philadelphia School of Medicine, in a body, have applied for and received the several appointments. We infer that the two schools will be consolidated.

Drs. Hun and Dean have resigned in the Albany Medical College. Prof. Howard Townsend unites Physiology with Materia Medica; and Prof. Quackenbush, Medical Jurisprudence with Obstetrics.

Dr. J. G. Howard has resigned the chair of Anatomy, in the Savannah Medical College, and is succeeded by Dr. W. R. Waring.

Dr. Giddings has returned to the Medical College of South Carolina, to fill the place of Prof. Gaillard, deceased.

Prof. L. P. Yandell has resigned the chair of Physiology, in the Medical Department of the University of Louisville. Dr. Yandell has long been connected with the Louisville University; first, as Prof. of Chemistry, and latterly, as the incumbent of the chair which he has just vacated. We understand that he intends leaving the city of Louisville, to return to Tennessee, where he formerly resided.

Dr. Thomas M. Markoe has been appointed Lecturer-adjunct to the Prof. of Surgery, and Dr. George T. Elliott, Jr., Lecturer-adjunct to the Prof of Obstetrics, in the College of Physicians and Surgeons in this city.

